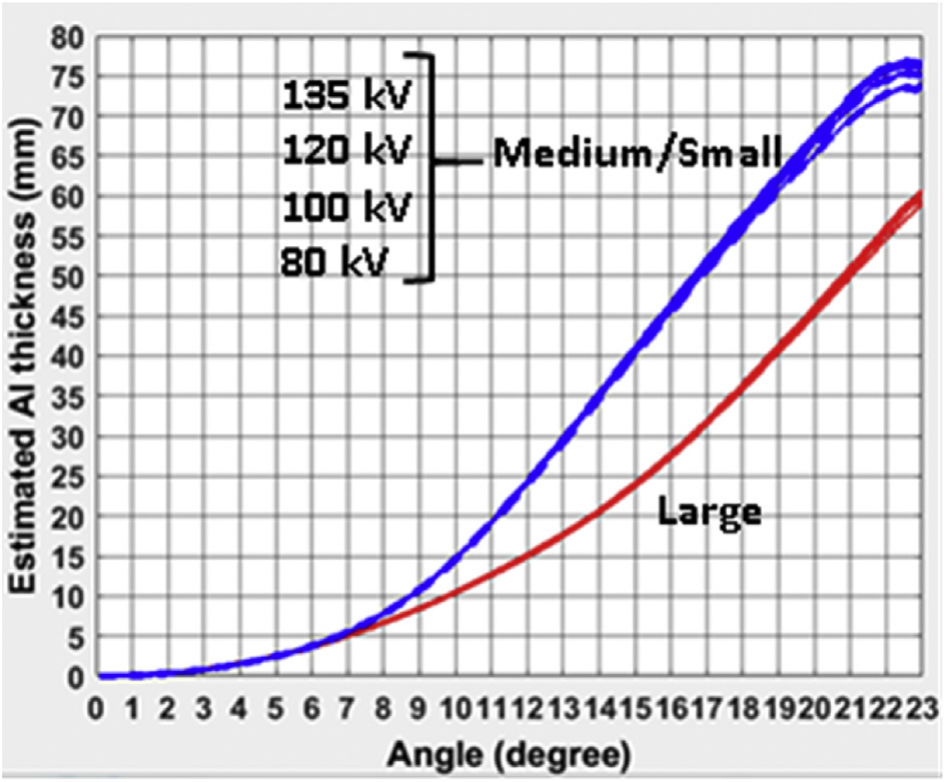# Data of CT bow tie filter profiles from three modern CT scanners

**DOI:** 10.1016/j.dib.2019.104261

**Published:** 2019-08-08

**Authors:** Kai Yang, Chun Ruan, Xinhua Li, Bob Liu

**Affiliations:** a Department of Radiology, Massachusetts General Hospital, Boston, MA 02114, USA; b Department of Radiology, Brigham and Women's Hospital, Boston, MA 02115, USA

**Keywords:** Computed tomography (CT), Beam filtration, Bowtie

## Abstract

As one of the key hardware components in Computed Tomography (CT) scanners, a bowtie filter reduces unnecessary radiation dose to the peripheries of a patient and equalizes radiation signal to the detector. Knowledge of the exact profiles from different bowtie filters are critical to model the imaging process of CT scanners and to estimate patient dose. However, bowtie filter profiles remain proprietary to most CT vendors. The data of bowtie profiles reported here were directly measured using a solid-state linear-array detector from three modern CT scanners (GE Revolution, Philips iQon, and Toshiba Aquilion ONE Vision. The detailed method, including associated geometrical calibration and data validation, was previously published. This data article is mainly to present the updated bowtie profile data measured after our previous publication.

**Table:** Specifications Table

Subject area	*Physics*
More specific subject area	*Medical Physics*
Type of data	*Table, graph*
How data was acquired	*Measured with a linear-array solid-state x-ray detector (X-Scan 0.8f3-512; Detection Technology Inc., Espoo, Finland) from three modern CT scanners (*GE Revolution, Philips iQon, and Toshiba Aquilion ONE Vision)
Data format	*Calculated tabulated data from measurements, specifically presented as equivalent aluminum thickness at different bow-tie angle.*
Experimental factors	*Measured raw exposure data were corrected for angular response and gain differences between each detector pixel, after simultaneous geometrical calibration.*
Experimental features	*The linear detector was placed inside the CT gantry and x-ray intensity through an axial CT rotation was recorded at 0.*24 ms *per line with 0.*8 mm *spatial resolution. All the available bow-tie filters were measured under four different kVps (80,100,120,135/140).*
Data source location	*Massachusetts General Hospital, Brigham and Women's Hospital, Boston, Massachusetts, USA.*
Data accessibility	*Data is with this article.*
Related research article	Yang K, Li X, George Xu X, Liu B. Direct and fast measurement of CT beam filter profiles with simultaneous geometrical calibration. Med Phys. 2017; 44 (1):57–70. https://doi.org/10.1002/mp.12024. PubMed PMID: 28,102,951; PubMed Central PMCID: PMCPMC5543987.

**Table:** 

**Value of the data** • The data presented here are CT bowtie profiles, which were directly measured using a linear array detector with high spatial and temporal resolution. • The data can be valuable for researchers interested in CT dosimetry, CT system modeling and CT scanner evaluation, since bowtie filter profiles are typically proprietary information from most CT vendors. • The direct usage of the presented data is to set up computer simulation models (such as Monte Carlo simulation) for different CT scanners using the corresponding bowtie profiles.

## 1 Data

A total of seven different CT bowtie filter profiles are reported for three CT scanners: GE Revolution, Philips iQon, and Toshiba Aquilion ONE Vision. For each individual bowtie filter, the profiles were measured and reported for four different kVps (80,100,120,135/140), in the format of equivalent thickness of Aluminum as a function of the bowtie angle (fan angle). A total of three figures are provided for the bow tie filter profiles for each scanner model. The attached excel file provides numerical values of those profiles in three separate tables.

## 2 Experimental design, materials and methods

With a previously published method (1), x-ray beam transmission profiles were first measured with a solid-state linear-array detector (X-Scan 0.8f3-512; Detection Technology Inc., Espoo, Finland), which has a linear array of 1 x 640 pixels (size of 0.8 mm × 0.7 mm) and covers a linear length of 51.2 cm. With the combined simultaneous geometrical calibration and knowledge of x-ray spectrum from Tungsten anode, the measured transmission profiles were converted into equivalent thickness of Aluminum. In order to best demonstrate the relatively different shapes, the profiles shown in below graphs were generated from the measured results subtracted by the aluminum thickness at bowtie center (zero degree angle). Detailed numerical data is included in the supplemental excel file.

GE Revolution: three different bowtie filters were measured. As in the GE manual, “Large” is for Large Body and Cardiac Large. “Medium” is for Head, Medium Body, and Cardiac Medium. “Small” is for Small Head, Small Body, Cardiac Small, Pediatric Head, and Pediatric Body.

**Figure:**
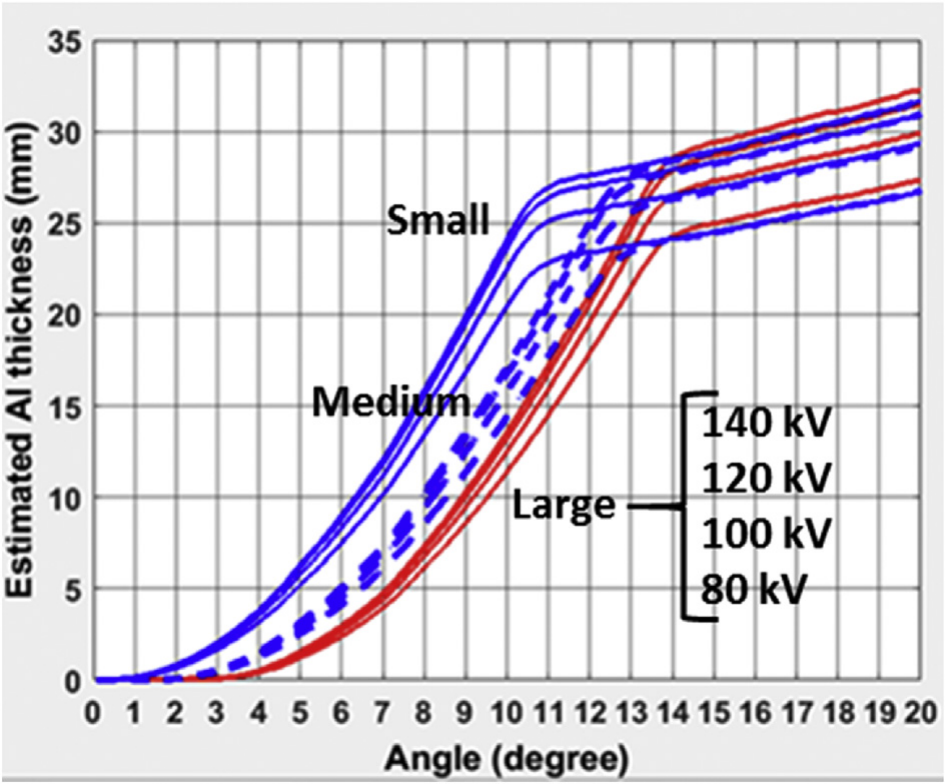

Philips iQon: one filter profile was measured and was identical for all scan modes of Infant and Adult Head/Adult Body, Cardiac, and Infant Body.

**Figure:**
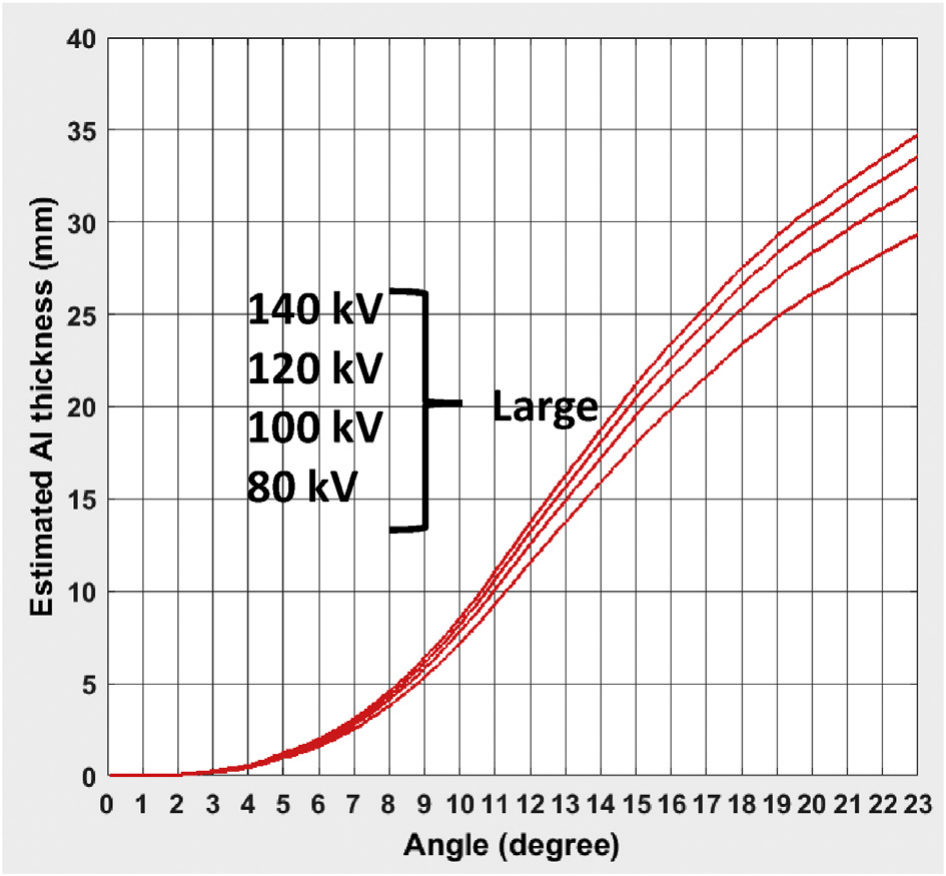

Toshiba Aquilion ONE Vision: three different bowtie filters were measured. Large (L) and Medium (M) have identical Al thickness at bowtie center but different profiles. Medium (M) and Small (S) have identical shape, but different Al thickness at bowtie center.

**Figure:**